# Long term cerebral and vascular complications after irradiation of the neck in head and neck cancer patients: a prospective cohort study: study rationale and protocol

**DOI:** 10.1186/1471-2377-14-132

**Published:** 2014-06-19

**Authors:** Joyce Wilbers, Arnoud C Kappelle, Roy PC Kessels, Stefan CA Steens, Frederick JA Meijer, Johannes H Kaanders, Roy AM Haast, Laura E Versteeg, Anil M Tuladhar, Chris L de Korte, Hendrik HG Hansen, Frank J Hoebers, Willem Boogerd, Erik D van Werkhoven, Marlies E Nowee, Guus Hart, Harry Bartelink, Lucille D Dorresteijn, Ewoud J van Dijk

**Affiliations:** 1 Department of Neurology, Radboud University Nijmegen Medical Center, Donders Institute for Brain, Cognition and Behaviour Centre for Neuroscience, PO box 9101, 6500 HB Nijmegen, The Netherlands; 2 Department of Radiation Oncology Radboud University Nijmegen Medical Center, PO box 9101, 6500 HB Nijmegen, The Netherlands; 3 Department of Radiology, Radboud University Medical Center, PO box 9101, 6500 HB Nijmegen, The Netherlands; 4 Department of medical psychology, Radboud University Medical Center, Donders Institute for Brain, Cognition and Behaviour, Radboud University Nijmegen, 6500 HE Nijmegen, The Netherlands; 5 Department of Neurology, Netherlands Cancer Institute/ Antoni van Leeuwenhoek Hospital, PO box 90203, 1006 BE Amsterdam, The Netherlands; 6 Department of Radiotherapy, Netherlands Cancer Institute, Antoni van Leeuwenhoek Hospital, PO Box 90203, 1006 BE Amsterdam, The Netherlands; 7 Department of Biometrics, Netherlands Cancer Institute/ Antoni van Leeuwenhoek Hospital, PO box 90203, 1006 BE Amsterdam, The Netherlands; 8 Maastricht University Medical Center, Department of Radiation Oncology (MAASTRO clinic), GROW School for Oncology and Developmental Biology, PO box 3035, 6202 NA Maastricht, The Netherlands; 9 Department of Neurology, Medisch Spectrum Twente, PO Box 50000, 7500 KA Enschede, The Netherlands; 10 Medical Ultrasound Imaging Center (MUSIC), Department of Radiology and Nuclear Medicine, Radboud University Medical Center, Geert Grooteplein zuid 10, 6525 GA Nijmegen, The Netherlands

**Keywords:** Head and neck cancer, Cerebrovascular disease, Carotid artery, Radiation vasculopathy

## Abstract

**Background:**

Successful treatment options for cancer result in more young long-term survivors prone for long-term complications. Carotid artery vasculopathy is a potential long-term complication after radiotherapy of the neck, resulting in cerebrovascular events and probably deficits in cognitive and motor functioning. Better insight into the underlying pathofysiology of radiotherapy induced carotid artery vasculopathy is needed for prognostic purposes and to develop preventive strategies.

**Methods/Design:**

The current study is a prospective cohort study on the long-term cerebral and vascular complications after radiotherapy of the neck, in 103 patients treated for head and neck cancer, included in our study database between 2002 and 2008. Baseline protocol (before radiotherapy) included screening for cerebrovascular risk factors and intima media thickness measurement of carotid arteries by ultrasonography. Follow-up assessment more than 5 years after radiotherapy included screening of cerebrovascular risk factors, cerebrovascular events, neurological examination with gait and balance tests, extensive neuropsychological examination, self-report questionnaires, ultrasonography of the carotid arteries with measurement of intima media thickness and elastography, magnetic resonance imaging of the brain and magnetic resonance angiography of the carotid arteries.

**Discussion:**

The current study adds to the understanding of the causes and consequences of long-term cerebral and vascular changes after radiotherapy of the neck. These data will be helpful to develop a protocol for diagnostic and preventive strategies for long-term neurological complications in future head and neck cancer patients with anticipated radiotherapy treatment.

## Background

Successful treatment options for cancer have resulted in more long-term survivors, prone to long-term complications of chemotherapy and radiotherapy (RT). A well-known long-term complication of RT of the neck is carotid artery vasculopathy, which ultimately can lead to stroke [1,2].

Apart from acute neurological complications, like a cerebral infarction, evidence from daily clinical practice shows more subtle, slowly progressive deficits in cognitive and motor functioning. These long-term cerebral and vascular complications in young survivors of cancer are a serious potential problem that hamper reintegrating in social functioning and work. The longer survival stresses the need for a long-term follow-up study that assesses the long-term complications of RT induced carotid artery vasculopathy. To date, no such studies exist to the best of our knowledge.

We hypothesize that RT of the neck accelerates carotid atherosclerosis with secondary development of cerebral vascular lesions. These vascular cerebral changes are subsequently related to cognitive and motor functioning with a major impact on activities of daily social functioning. A better understanding of the underlying pathophysiology of this RT induced vasculopathy is needed for prognostic purposes and can be used to develop strategies that prevent further progression and thereby reducing the risk of future stroke and neurological impairments.

The current study aims to investigate four questions. The first question focuses on the relation between RT and long-term carotid artery-wall changes. We hypothesize that irradiation induces premature atherosclerosis which is different from more common atherosclerosis due to classic cerebrovascular risk factors. We therefore study the location and composition of post-irradiation vessel-wall changes with advanced imaging techniques; plaque and Intima Media Thickness (IMT) analysis on ultrasound with innovative vessel-wall elastography and magnetic resonance imaging/angiography (MRI)/(MRA) [3-5]. The second question examines the relation between RT and long-term cerebral vascular changes. In the general population the prevalence of silent brain infarcts (SBI) is up to fivefold higher than that of acute stroke [6]. SBI are related to impaired cognitive functioning, dementia, stroke and gait and balance disorders [7]. Carotid artery pathology increases the risk of SBI [8]. To assess this, all study patients underwent MRI of the brain to analyze silent brain infarcts and related pathology like white-matter lesions and cerebral atrophy.

The third question addresses the relation between RT and clinical outcome measures (motor and cognitive functioning, as well as quality of life; QoL). Silent brain infarcts are associated with gait disturbances, decline in cognitive function and increased risk of developing dementia [9-11]. We performed quantitative gait/balance analysis and an extensive neuropsychological assessment in all patients. Finally, we want to study the relation between patient and treatment baseline characteristics and the development of vasculopathy, in order to find high risk patients that can benefit from preventive strategies.

## Methods

The current study is a prospective cohort study that aims to investigate the frequency, pathophysiology and long-term outcome of radiotherapy induced vasculopathy. The Medical Ethics Review Committee region of Arnhem-Nijmegen approved the study (NL 41008.091.12).

### Patients

A prospective cohort study of head and neck cancer patients in two centers of the Netherlands (the Netherlands Cancer Institute/Antoni van Leeuwenhoek Hospital (NCI/AvL), Amsterdam and the Radboud University Medical Center Nijmegen) was initiated.

At baseline, patients were eligible for inclusion if they received RT of the neck because of a T1/T2 (N0M0) laryngeal carcinoma, T1/T2 (N0M0) parotid carcinoma/pleomorphic adenoma, T1/2 (N1/2 M0) naso/oro/hypopharynxcarcinoma or non-Hodgkin/Hodgkin lymphoma. Originally, the study was designed as an open-label multicentre Prospective Randomized Open Blinded Endpoint (PROBE) study to assess the effect of an HMG-coA reductase inhibitor (atorvastatine) on carotid IMT in the first 2 years after irradiation of the neck. Because of dwindling accrual the study was redesigned to a prospective cohort study. The initial exclusion criteria were: a history of cerebrovascular disease, pregnancy or breast-feeding, ongoing treatment with an HMG-coA reductase or cytochroom P450 inhibitor, active liver disease or > 3 times the upper limit of serum transaminases, 5 times the normal level of creatine phosphokinase, serum cholesterol > 7 mmol/L and a life expectancy < 2 years. All patients gave written informed consent.

### Controls

The IMT measurements will be compared to the baseline measurements and the elastography measurements will be compared to an age and sex matched control group of patients who had a stroke and at least one of the following cerebrovascular risk factors: hypertension, diabetes, smoking, hypercholesterolemia and atrial fibrillation. The cognitive performances of all patients will be compared to available normative reference values (adjusted for age and education level). Finally, the frequency of silent brain infarctions and white matter lesions will be compared to the frequency in patients without RT of the neck, as described in prior population based studies [12,13].

### Baseline

Patients were assessed at baseline (before RT) for height, weight and blood pressure, underwent a neurological and laboratory (C-reactive protein) examination and the following cerebrovascular risk factors were assessed:

1. Cigarette smoking. 2. Hypertension, defined as using antihypertensive medication or blood pressure > 130/80 mmHg. 3. Diabetes mellitus, defined as using anti-diabetic medication or a non-fasting serum glucose >11.1 mmol/L. Medication prescription was checked with the pharmacy database. 4. Hypercholesterolemia, defined as serum total cholesterol > 6.5 mmol/L. 5. Obesity, defined as Body Mass Index (BMI) > 30 kg/m^2^.

#### Carotid ultrasound

In patients with laryngeal or hypopharyngeal carcinoma IMT was measured on the Common Carotid Artery (CCA), according to the RT-field. In patients with other tumors IMT was measured in both the CCA and the Internal Carotid Artery (ICA), also according to the RT- field.

IMT was measured with a linear array transducer (iU22 Philips NZE172 probe: L17-5, Hewlett Packard, Sonos 2000, probe: 7.5/5.5, ALOKA 5000 and G.E Logiq E9). IMT pictures were digitally stored on the ultrasound machine or printed in case digital storage was not available. Printed images were then scanned at high resolution and interpolated (using Matlab 2010a) to obtain the same resolution as the digitally stored images. IMT was automatically measured by one blinded investigator (JW), using QLAB (Version 4.2.1, Philips). An edge-detection algorithm identified the lumen-intima and the media-adventia interface within a region of interest (selected by the operator) over a maximum of 10 mm long segment and calculated the average thickness.

### Follow-up > 5 years after radiotherapy

At follow-up all patients underwent a structured program (Table 1), consisting of assessment of demographics and life style, medical history and medication use, fasting laboratory investigation, electrocardiogram (ECG), physical and neurological examination, extensive neuropsychological assessment battery (NPA), carotid artery ultrasound (Intima Media Thickness measurement and elastography), MRI/MRA of the brain and carotid arteries, gait and balance analysis and self-report questionnaires.

**Table 1:** Schedule of assessments

**Assessment**	**Baseline**	**Follow-up**
**Demographics**		
Worksituation		X
Education		X
Marital status		X
Social/living status		X
Oncologic diagnose	X	X
**Radiotherapy**		
Dosis Total (Gy)	X	X
Dosis per fraction (Gy)	X	X
Number of fractions	X	X
Bilateral/unilateral	X	X
Dose on common carotid artery	X	X
Dose on internal carotid artery	X	X
**Medical history**		
Cerebrovascular events	X	X
Cerebrovascular risk factors	X	X
Cardiovascular events		X
Hypertension	X	X
Hypercholostrolemia		X
Smoking	X	X
Diabetes		X
Family history of cerebrovascular disease		X
Obesity		X
Migraine		X
**Medication use**		
Aspirin		X
Oral anticoagulance		X
Antihypertensive medication		X
Statin		X
Anti-diabetic medication		X
**Physical examination**		
Length and weight		X
Waist circumference		X
Blood pressure	X	X
BMI		X
**Neurological examination**		
Cranial nerves		X
Muscle strength		X
Coordination		X
**Mobility**		
Time up and Go/Tinetti		X
**ECG**		
Paroxysmal atrial fibrillation		X
Left ventricle hypertrophia		X
Ischemia		X
**Fasting laboratory investigation**		
Glucose		X
Total cholesterol		X
**Ultrasonography**		
IMT	X	X
Elastography		X
**Neuropsychological Examination**		
*Global cognitive functioning*		
Minimal Mental State Examination (MMSE)		X
Frontal Assessment Battery (FAB)		X
*1. Episodic Memory*		
Rey Auditory Verbal Learning Test (RAVLT)		X
*2. Working memory*		
Paper and Pencil Memory Scanning Tasks		X
Digit Span test		X
*3. Executive functioning*		
Stroop Color-Word Task		X
Trail Making Test (TMT)		X
Brixton Spatial Anticipation Test		X
*4. Attention*		
Verbal Series Attention Test(VSAT)		X
*5. Fluency Language*		
Letter fluency		X
Animal fluency		X
*6. Speed information processing*		
TMT A		X
Stroop test (mean score Parts I and II)		X
Letter Digity Substitution Task		X
**Questionnaires**		
*Depressive symptoms*		
Structured questionnaire depressive symptoms		X
Mini International Neuropsychiatric Interview (MINI)		X
Hospital Anxiety and Depression Scale (HADS)		X
Center of Epidemiological Studies Depression scale		X
*Fatigue*		
CIS 20 R		X
*Health related Quality of Life*		
Short form36		X
*Others*		
Structured questionnaire about sleep disorders		X
Subjective memory complaints		X
**MRI**		
Brain (T1, T2, FLAIR)		X
Carotid arteries (TOF, PCA, T1, T2, PD)		X

#### Demographics and life style

Standardized questions on demographics (age at radiotherapy, age at follow-up, sex) were administered. Education was classified using seven categories; one being less than primary school and seven reflecting an academic degree. Type of work, working situation, marital status and living conditions were asked using a structured interview.

#### Medical history and medication use

Standard questionnaires about medication use (carbaspirin calcium, oral anticoagulance, antihypertensive medication, statin, antidiabetic medication), cerebrovascular risk factors (hypertension, hypercholesterolemia, smoking, diabetes, family history, obesity, migraine), vascular diseases in the past (stroke, cerebral bleeding, acute myocard infarction, atrial fibrillation, angina pectoris, peripheral arterial disease) were asked by one of the investigators (JW). If patients had had an ischemic stroke (IS) or Transient Ischaemic Attack (TIA), detailed medical information was collected from their neurologist and reassessed by one neurologist (EvD).

#### Fasting laboratory investigation

Fasting blood samples were taken. Analysis included glucose and total cholesterol.

#### Electrocardiogram (ECG)

An ECG was performed and evaluated with a standardized assessment by an experienced cardiologist. Outcome measures were (paroxysmal) atrial fibrillation, left ventrical hypertrophia and signs of ischemia (pathologic Q’s, ST depression or elevation).

#### Physical and neurological examination

Height and weight were measured and body mass index (BMI) was calculated. The maximal weight circumference was measured in standing position, between the lowest rib and the iliac crest, at the end of normal expiration [14]. Blood pressure was measured on the right and left side. All patients underwent a standardized neurologic examination by an experienced neurologist (JW), consisting of assessment of functioning of cranial nerves, muscle strength and cerebellar functions.

#### Assessment of gait and balance

We used a widely used version of the Tinnetti with 17 items: 9 items for body balance (score 0-16) and 8 for gait (score 0-12), with a maximum score of 28 [15]. It grades balance while sitting, standing with eyes open and closed, nudging and turning, gait initiation, stride length and width and symmetry. Functional mobility was classified by using the widely-used Timed Up-and-Go (TUG) test which is a timed test during which the participant is asked to rise from a standard armchair, walk 3 m, turn, walk back and sit down again [16]. Each participant performed the test three times.

#### Neuropsychological assessment

A trained psychologist administered an extensive neuropsychological test battery that covered 6 cognitive domains; episodic memory, working memory, executive functioning, attention, fluency language and speed of information processing. The Mini Mental State Examination (MMSE) [17] and the Frontal Assessment Battery (FAB) [18] were used as a screening of overall cognitive function and orientation. Episodic memory was assessed using the five-trial Dutch version of the Rey Auditory Verbal Learning Test (RAVLT) in which the ability to acquire and retain new verbal information was measured, immediately and after a delay [19]. We administered the Paper and Pencil Memory Scanning Task [20] and the Digit Span test [21], both forwards and backwards, to assess working memory. Executive functioning was assessed using the Stroop Color-Word Task (Stroop interference score; i.e. Part III/mean(Part I and II)) [22], the Trail Making Test (TMT) [23] ratio score (part B/part A) and the Brixton Spatial Anticipation Test (Brixton) [24,25]. Attention was assessed with the Verbal Series Attention Test(VSAT) [26]. To evaluate verbal fluency two tasks were used. First, a semantic fluency task [27] was administered in which participants had to name as many animals as possible within 60 seconds followed by as many professions within 60 seconds. Second, a letter fluency task [28] was used in which as many words beginning with a given initial letter (D-A-T) had to be generated within 60 seconds. Speed of information processing was evaluated by using three tasks; the Stroop test (mean score Parts I and II), the TMT Part A and the Symbol Digit Substitution Task [29], which is a modified version of the Symbol Digital Modalities Task.

#### Carotid artery ultrasound - Intima Media Thickness measurement

The measurement of IMT at follow-up 7 years after RT was exactly the same as described at baseline.

#### Non-invasive carotid ultrasound elastography – imaging protocol

Following IMT measurements, the distal CCA and proximal ICA at both sides were examined in a longitudinal and transverse imaging plane using a Medison Accuvix V10 ultrasound system (Samsung Medison Seoul, Republic of South Korea) equipped with an L5-13 linear array transducer (fc = 8.5 MHz). Firstly, longitudinal recordings were acquired. After optimum visualization of the cross section with the highest amount of luminal narrowing, an imaging mode called radial zone mode was turned on, which enabled us to perform angle compounding and to store raw ultrasound radiofrequency (RF) data (fs = 61.6 MHz). RF data were acquired at three insonification angles (0°, 20° and -20°) for 3 seconds at a frame rate of at least 129 Hz (43 Hz/angle). Insonification angles were relative to the unsteered ultrasound beam direction and changed sequentially before every new ultrasound image frame. Depending on the heart rate, this resulted in the recording of at least two complete cardiac cycles. After the described measurement, the systolic and diastolic blood pressures in the brachial artery were determined using a sphygmomanometer (Littmann® Classic II S.E.; 3 M, St Paul, MN, USA). The pressure values were used to normalize the distensibility and strain results as explained later, since the level of strain is not only related to tissue composition but also to the pressure differential between the subsequently acquired frames. Above described steps were repeated during imaging in the transverse plane. Location of transverse image acquisition was chosen at the vessel wall displaying the thickest IMT. The distance between this cross-section and the carotid bulb was used as an anatomical reference point. Subsequently, this protocol was repeated for the internal carotid artery. The entire measurement series was completed within 30 minutes. Raw RF data were subsequently analyzed to determine distensibility and strain using in-house written elastography software (MATLAB 2010b, the Mathworks, Natick, MA, USA). Detailed description of this can be found in Additional file 1.

#### MRI protocol

The MRI studies were performed on a 3 Tesla MR-scanner (Skyra, Siemens Erlangen). The scanning protocol included T1, T2 and T2 Fluid Attenuated Inversion Recovery (FLAIR) transversal sequences of the brain. The MRI scanning protocol of the carotids included a 3 dimensional time of flight (TOF), Phase Contrast Angiography (PCA), sagittal oblique T2 of the carotid bifurcation, transversal T1, T2 and proton density (PD) sequences. Finally, a maximum intensity projection (MIP) TOF and PCA was reconstructed. The complete scanning protocol took approximately 60 minutes. All MRI studies will be scored by two blinded experienced neuroradiologists (SS and FAM).

#### Additional self-report questionnaires

For the assessment of anxiety and depression we used the Hospital Anxiety and Depression Scale (HADS) and the Center for Epidemiologic Studies Depression Scale (CES-D) [30]. The Checklist on Individual Strength (CIS-20R) was used to assess fatigue [31]. The overall health status (quality of life) was assessed with the Short Form 36 Health Survey (SF-36) [32]. Patients were asked for sleeping problems and their employment status in the period before and the period after radiotherapy. Two questionnaires were filled in with a doctor (JW): the assessment of Subjective Memory Complaints (SMC) [10] and the Mini International Neuropsychiatric Interview (MINI) [33].

### Outcome events

#### Imaging outcomes

Firstly, on ultrasonography, the IMT of the carotid artery 6 years after RT will be compared with the IMT at baseline. Furthermore, the strain in the vessel wall and plaque 6 years after RT will be measured and compared with a control group. Secondly, on MRI/A of the carotid arteries the degree of stenosis, location and when possible composition of vessel wall changes (lipids, fibrosis, hemorrhage) will be scored, according to an in house developed protocol.

All MRIs of the brain will be scored semi-quantitatively by two neuroradiologists separately for white-matter lesions (WML), brain atrophy and (silent) brain infarctions using validated scales [34-36] and in case of disagreement a consensus meeting will be held.

Furthermore, total white matter lesion volume will be calculated by a prior published, in-house developed, validated technique [37]. In short, WML are defined as hyperintensive lesions on FLAIR MRI without corresponding cerebrospinal fluid like hypo-intense lesions on the T1 weighted image. Gliosis surrounding lacunar and territorial infarcts is not considered to be WML. Finally, brain volumetry will be done to measure total white and grey matter volumes. Grey and white matter tissue and cerebrospinal fluid probability maps will be computed by using a six class segmentation tool in Statistical Parametric Mapping Software (http://www.fil.ion.ucl.ac.uk/spm) (SPM 8) on the T1 MPRAGE images. Total grey and white matter volumes are calculated by summing all voxel volumes belonging to the tissue class. Total brain volume is taken as the sum of total grey and total white matter volume. Co-registration parameters of the FLAIR image to the T1 image are computed (SPM8 mutual information co-registration) and used to bring both the FLAIR and WML segmentation images into the subjects (anatomical) reference frame. Transformed images will visually be checked for co-registration errors. Subsequently, the WML segmentations are re-sampled to and combined with the white matter maps to yield to a WML map (the intersection of WML and white matter) and NAWM map (the complement of WML in white matter) in the T1 reference space.

#### Clinical outcomes

Clinical outcome measures will be: cerebrovascular events, domain scores (mean standardized z-scores compared to reference values) of objective cognitive functioning on NPA, subjective memory complaints, depressive symptoms, fatigue and gait or balance disturbances.

### Statistical analysis

Analysis of continuous variables will be done with Student’s *t* test or analysis of (co)variance or in case of skewed distributions which cannot be normalized by log transformation corresponding nonparametric tests will be used. Chi-squared test will be used for analysis of categorical variables and logistic regression analyses will be used to adjust for potential confounding factors.

## Results

**Figure 1:**
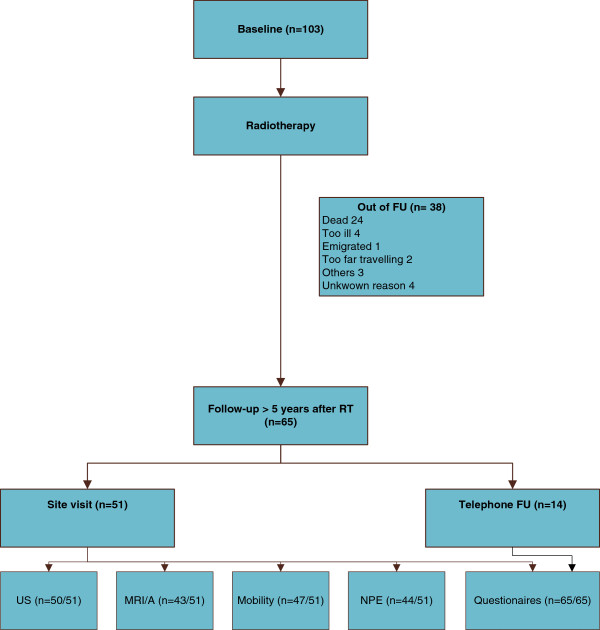
Flow chart.

103 patients who had sought medical attention at one of the two University Medical Centers between 2002 and 2008 fulfilled inclusion and exclusion criteria for our study. The number of patients lost to follow-up is reported in Figure 1. 51 patients have been seen for the follow-up protocol more than 5 years after RT between November 2012 and November 2013. Another 14 patients underwent a telephone follow-up. Characteristics of our population at baseline and follow-up are reported in Table 2.

**Table 2:** Patient and treatment related characteristics at baseline and FU

**Characteristic**	**n = 65**
**Demografics**	
Male (%)	60
Age at baseline, years (mean, SD)	54.3 (13.3)
Follow-up post RT, years (mean, SD)	6.7 (1.2)
Age at FU, years (mean, SD)	61.2 (13.4)
**Cancer diagnosis (%)**	
Larynxcarcinoma	45
Parotidcarcinoma	14
Pleiomorphic parotid adenoma	15
Nasopharynxcarcinoma	2
Oropharynxcarcinoma	17
Hypopharynxcarcinoma	2
Lymphoma	6
**Radiotherapy (%)**	
Bilateral	63
Unilateral	37
**CV risk factors at baseline (%)**	
Smoking	
Current smoker or stopped < 3 years ago	51
Former smoker or stopped > 3 years ago	23
Never smoked	25
Hypertension	26
Diabetes mellitus	5
Hypercholesterolemia	9
Obesity	8
**Number CV risk factors at baseline‡ (%)**	
0	32
1	43
≥2	25
**CV risk factors at FU (%)**	
Smoking	
Current smoker or stopped < 3 years ago	22
Former smoker or stopped > 3 years ago	59
Never smoked	20
Hypertension	69
Diabetes mellitus	11
Hypercholesterolemia	39
Obesity	12
Migraine	11
Family history of CV diseases	20
**Vascular diseases in the past (%)**	
Angina Pectoris	5
Atrial Fibrillation	11
Acute Myocardial Infarction	3
Peripheral arterial disease	2
**Weight circumference, cm (mean, SD)**	95.5 (16.6)
Number CV risk factors at FU* (%)	
0	9
1	28
≥2	63

‡Cerebrovascular (CV) risk factors at baseline consists of hypertension diabetes, hypercholostrolemia, overweight and current smoking.

*Cerebrovascular (CV) risk factors at FU consists of hypertension, diabetes, hypercholesterolemia, overweight, current smoking, migraine, family history of vascular disease and Atrial Fibrillation (AF). NA = Not Answered.

## Discussion

The number of cancer survivors is still growing and long-term complications after cancer treatment are becoming a serious problem for the society. However, prospective cohort studies with a long follow-up period after RT of the neck are scarce. More understanding about the underlying pathophysiology of RT induced vasculopathy is needed to develop preventive strategies, such as the use of a statin or thrombocyt aggregation inhibitors. These long-term complications of RT induced carotid artery vasculopathy are not studied in prior studies, because of relatively small patient groups and short follow-up period.

We therefore performed the current prospective cohort study, to investigate the cerebral and vascular long-term complications after RT of the neck. Strong elements of our study are the unique population and study design. This cohort is not only the largest, but also has the longest follow-up period (more than 5 years after RT). Furthermore, we performed a complete assessment with radiological and clinical outcome measurements. We use newly developed innovative ultrasonography and MRI techniques, to investigate the underlying pathofysiology of RT induced vasculopathy. Also, we examined cognitive function with sensitive neuropsychological tests covering all cognitive domains, rather than just relying on cognitive screens aimed at the detection of dementia.

We feel that the current study helps us in the understanding of the causes and consequences of long-term cerebral and vascular changes after RT of the neck. These data will be the source to make a protocol for diagnostic and preventive strategies for long term neurological complications in future HNC patients.

## Competing interests

The authors declare that they have no competing interests.

## Authors’ contributions

JW/LV/RH contribution to conception and design; acquisition of data; involvement in drafting the manuscript; final approval of the version to be published. JW/LV/RH/RK/CK/SS/FAM/AK/EvD/RK/FH/WB/EW/LD contribution to conception and design; revising the manuscript critically; final approval of the version to be published. All authors read and approved the final manuscript.

## Pre-publication history

The pre-publication history for this paper can be accessed here:

http://www.biomedcentral.com/1471-2377/14/132/prepub

## Supplementary Material

Additional file 1 **Non-invasive carotid ultrasound elastography - data analysis [**[38]**-**[40]**].** Click here for file
