# Supplementary material for: Long term cerebral and vascular complications after irradiation of the neck in head and neck cancer patients: a prospective cohort study: study rationale and protocol
Source: BMC Neurol. 2014 Jun 19;14:132. doi: 10.1186/1471-2377-14-132 (PMC4077148; doi:10.1186/1471-2377-14-132)
Supplement: Additional file 1 — Non-invasive carotid ultrasound elastography - data analysis [[38]-[40]]. [file 1471-2377-14-132-S1.doc]

**Additional file**

## Non-invasive carotid ultrasound elastography - data analysis

Briefly, RF data were pre-processed before calculation of the strain and distensibility values. Pre-processing consisted of a correction for grating lobes (artifacts that arise when performing large angle ultrasound beam steering) by band-pass filtering . Secondly, custom algorithms were applied to determine the vessel borders (adventitia-media [outer wall] and intima-lumen [inner wall]), regions of interest, vessel diameter changes over time and cardiac cycles (between two time points corresponding to maximum diameter). The degree of stenosis was calculated using the European Carotid Surgery Trial (ECST) method (1998) after defining the original lumen area.

Distensibility

Distensibility for both the inner and outer vessel walls was acquired using the values matching the maximum and minimum vessel diameter (i.e. distension) per cardiac cycle:

| 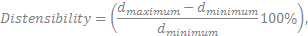 | ( 1 ) |
| --- | --- |

where d_maximum_ and d_minimum_ are equal to the values of the maximum and minimum diameter respectively.

Strain

Deformation of the tissue within the pre-defined ROIs was estimated between each sequential frame during the cardiac cycles. As described by Hansen et al. (2012), axial and lateral (along and perpendicular to the transmitted ultrasound beam, respectively) displacement of the tissue within the ROI were estimated for every 12.5 µm in the axial direction and 200 µm in the lateral direction, per beam steering angle. This was done using course-to-fine 2D cross-correlation displacement estimation in four iterative steps (see table 1 for parameter settings). Envelope data (Hilbert transformed RF data) were used during the first iteration step and RF data during the following steps.

Table 1 - Parameter settings displacement analysis

| **No. of iterations** | | | 4 |  |  |  |
| --- | --- | --- | --- | --- | --- | --- |
| **No. of course-to-fine correlations** | | | 3 |  |  |  |
| **Median filter** | | | 9x9 |  |  |  |
|  | | |  |  |  |  |
| **Window properties** | | |  |  |  |  |
| **Size** | | | **Iteration 1** | **Iteration 2** | **Iteration 3** | **Iteration 4** |
| *Pre-deformation* | | Axial points | 100 | 50 | 25 | 25 |
|  | | Lateral RF-lines | 3 | 3 | 3 | 3 |
| *Post-deformation* | | Axial points | 150 | 150 | 150 | 150 |
|  | | Lateral RF-lines | 9 | 9 | 9 | 9 |
| **Overlap** | Axial | | 80% | 80% | 80% | 80% |
|  | Lateral | | 67% | 67% | 67% | 67% |
| **Shape** | | | Rectangular | Free-shape & | Free-shape & | Free-shape & |
|  | | |  | Rectangular | Rectangular | Rectangular |
|  | | |  |  |  |  |
| **Course-to-fine correlation** | | | Yes | Yes | Yes | No |
| **Aligning** | | | No | No | No | Yes |

Axial displacement estimates of the 0° beam steering angles corresponded to the vertical displacement in the tissue, while the axial displacement estimates from the other two beam-steering angles (-20° and 20°) were combined to derive the lateral displacement, using:

| 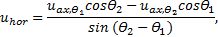 | ( 2 ) |
| --- | --- |

where u_hor_ is lateral displacement and u_ax,ϴ1_ and u_ax,ϴ2_ are estimated axial displacements of the beam-steered data at -20° and 20°, respectively. Both the lateral and the axiall displacements were cumulated over the cardiac cycle. Next, the radial and circumferential displacements were derived by combining the axial and lateral displacement components with respect to the lumen center. The lumen center was defined as the center of the area within the inner boundary (intima-lumen) of the ROI. Radial and circumferential displacements were transformed to radial and circumferential strain using 2-D least squares estimators . In case of longitudinal recordings, axial strain values were acquired by transforming the 0° axial displacement estimates. Multiple beam-steered data were used to obtain lateral displacements for ROI tracking.

Ultimately, distensibility and strain values were normalized for differences in blood pressure between patients using:

| 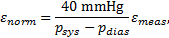 | ( 3 ) |
| --- | --- |

where ε_norm_ and ε_meas_ corresponded to normalized and measured strain/distensibility values, respectively, and p_sys_ and p_dias_ were systolic and diastolic blood pressures respectively.
